# Vaginal and reproductive organ preservation in trans men undergoing gender-affirming phalloplasty: technical considerations

**DOI:** 10.1093/jscr/rjab553

**Published:** 2021-12-28

**Authors:** Christopher J Salgado, Kerstin Yu, Maria J Lalama

**Affiliations:** Constructive Surgery Associates, Miami, FL 33145, USA; Constructive Surgery Associates, Miami, FL 33145, USA; Constructive Surgery Associates, Miami, FL 33145, USA

**Keywords:** phalloplasty, gender incongruence, transgender, cisgender, gender affirmation surgery

## Abstract

Gender affirmation surgeries are performed to decrease the patient’s dysphoria and improve quality of life. Preservation of the vaginal canal with reproductive organs is uncommon though becoming increasingly discussed in trans men. This series examines surgical complexities of vaginal and/or reproductive organ preservation in patients undergoing phalloplasty, highlighting considerations for safety and well-being. Two patients who underwent phalloplasty met inclusion and exclusion criteria for the study and were treated in accordance with World Professional Association for Transgender Health standards. We retrospectively reviewed patients’ medical records and extracted demographic data. Phallus and clitoral sensation, ability for penetrative intercourse and treatment of gender incongruence were assessed postoperatively. At an average of 22 months postoperatively, both flaps survived with tactile sensation two-thirds down the shaft, and the clitoris of both maintained erogenous sensation from stimulation. Gender incongruence was described to be reduced, and both patients were able to receive penetrative vaginal intercourse.

## INTRODUCTION

The surgical procedures performed for trans men during gender transition are dependent on the patients’ level of gender incongruence [1]. A combination of hormonal and mental health therapy may help a person feel more comfortable and pass in their identified gender, so surgery to treat their gender incongruence may not be required. However, gender affirmation procedures are often performed due to their beneficial effect on quality of life. Top surgery, or bilateral mastectomy, is often sought out as an initial procedure for trans men to alleviate their dysphoria, and this is occasionally performed in conjunction with the removal of their reproductive organs [2]. This combination procedure requires the support of two distinct mental health professionals recommending the patient undergo the procedure [3]. More frequently, a hysterectomy and oophorectomy are performed separately with some patients opting for egg harvest and preservation later. Removal of the uterus should include the cervix to eliminate risk of cervical cancer and continued screening with a gynecologist, something usually not preferred by trans men.

The vaginal canal may also be removed to decrease dysphoria but is not often done since it requires the skill of a trained urogynecologist, gynecology oncologist, reconstructive urologist or trained gender surgeon. The vaginal canal may be removed during a metoidioplasty procedure or phalloplasty where the urethra is either lengthened to the distal-most aspect of the metoidioplasty or diverted to the perineal region as a perineal stoma [4, 5, 6].

In patients who desire a phalloplasty procedure and do not request removal of the vaginal canal with urethral lengthening, the procedure is simplified. Since the urinary conduit does not require modification, the likelihood of otherwise common urethral complications is diminished. In these cases, urination will occur from the natal urethral meatus within the vagina. Preservation of the vaginal canal including their reproductive organs has been discussed in trans men patients who present for gender affirmation bottom phalloplasty, although it is extremely uncommon. Preservation of their reproductive organs requires routine screening of the ovaries, uterus and cervix to ensure that cancer or other pathology does not develop. These patients must be properly screened by a physician who is knowledgeable of this anatomy and associated pathology [7]. Usually, this would entail an evaluation by a gynecologist, which usually only increases a patient’s pre-existing dysphoria and is therefore uncommon.

Transgender male patients who present with gender incongruence related to the absence of a phenotypic male phallus may not be dysphoric due to the presence of their vaginal canal or reproductive organs. Significant reduction in their dysphoria resulting from a phalloplasty, which often visually conceals their female genital phenotype, may minimize their desire to remove their female reproductive organs, vulva and vagina. Additionally, they may not want to remove the vaginal canal so it can be used for intercourse. Keeping the canal with their reproductive organs may also allow them the ability to carry a baby to term in the future after discontinuing testosterone, accepting a relatively high risk of pregnancy loss [8]. Alternatively, they may desire to remove their uterus, fallopian tubes and vaginal canal, preserving their ovaries for possible egg harvest in the future and not at the time of hysterectomy to avoid the high costs associated with long-term egg storage in certified banks, a procedure not uncommonly performed. Egg harvest in these patients may be performed if desired along with an oophorectomy at that time.

The vaginal canal may also be chosen to be preserved in trans men to avoid surgical modification of their native urethral meatus to minimize the risk of urethral complications since the urethra is not lengthened and kept in the native vaginal canal. Alternatively, as in the case of a metoidioplasty, the urethral meatus may be lengthened using labia minora flaps and pedicled tissue from the anterior vaginal wall which will narrow the remaining vaginal canal. Regardless of reasoning, this procedure is becoming increasingly discussed, and there is a significant lack of discussion and direction in our current literature. The two cases presented highlight the surgical complexities related to the preservation of the vaginal canal and/or any of the trans man’s reproductive organs (ovaries, tubes, uterus and cervix) when performing a phalloplasty and the important preoperative, intraoperative and postoperative considerations that need to be considered for the safety and well-being of our trans community.

### Case 1

Patient is a 34-year-old trans man who is married to a cis-gender female. He previously underwent top surgery and later desired removal of his reproductive organs with preservation of his vaginal canal for two main reasons. Firstly, his dysphoria was unrelated to urinating in the standing position and more related to the absence of a phallus and his desire to use his phalloplasty for intercourse. In addition, he preferred to avoid the untoward sequelae associated with a vaginectomy and a urethral lengthening procedure [9]. Furthermore, the patient and his female spouse wanted to keep his vaginal canal for sexual intimacy. The patient had two letters from his mental health teams recommending him for surgery, both noting the patient’s desire to maintain his vaginal canal.

**Figure 1:**
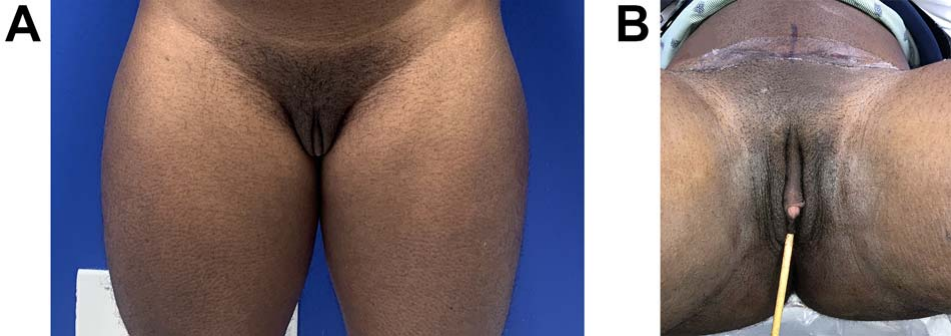
(**A** and **B**) A 34-year-old trans man prior to phalloplasty with latissimus dorsi flap and vaginal preservation. Previous double incision mastectomy for top surgery and a hysterectomy with oophorectomy had been done in the past.

**Figure 2:**
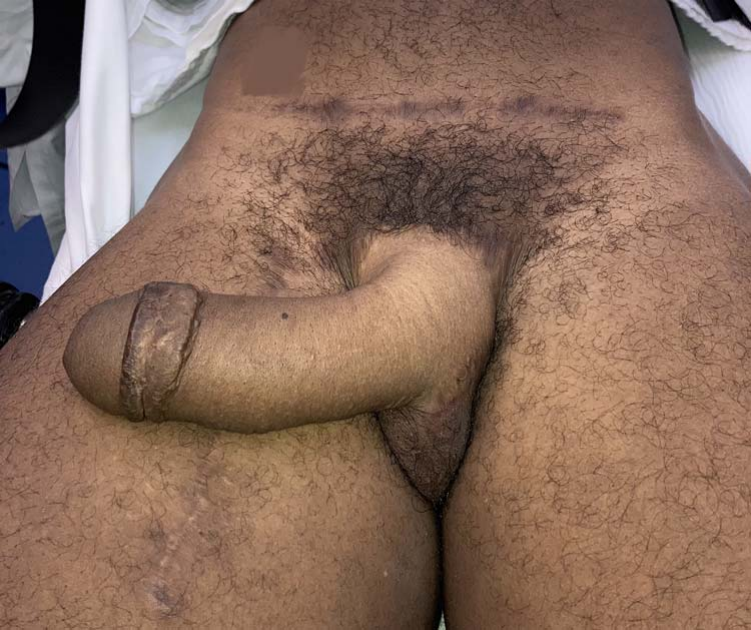
Patient 3 months postoperatively with viable neophallus using a split lateral latissimus dorsi myocutaneous flap without urethral lengthening for phalloplasty, glansplasty and scrotoplasty using labia majora flaps and preservation of the vaginal canal. One arterial and one venous anastomosis, and two neurorrhaphy with interposition nerve grafts to the ilioinguinal nerve and one clitoral nerve.

**Figure 3:**
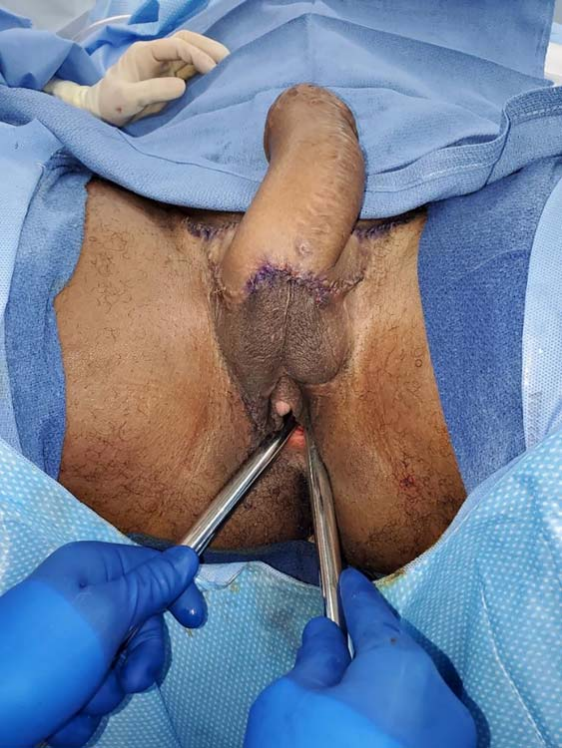
The vaginal canal was identified with Hegar dilators, allowing us to avoid injury while maintaining proper opening during the dissection.

**Figure 4:**
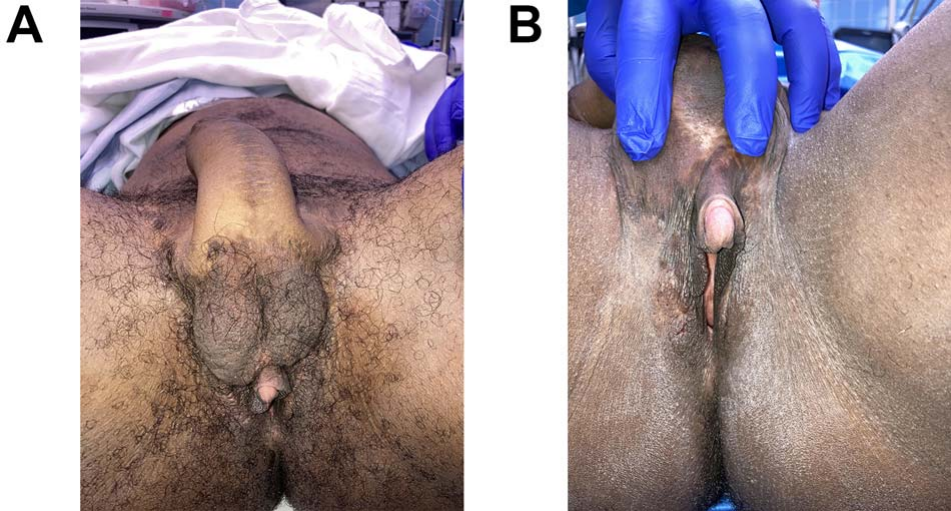
(**A** and **B**) Patient is shown 4 months following insertion of silicone testicular implants. Intact vaginal canal posterior to clitoris.

**Figure 5:**
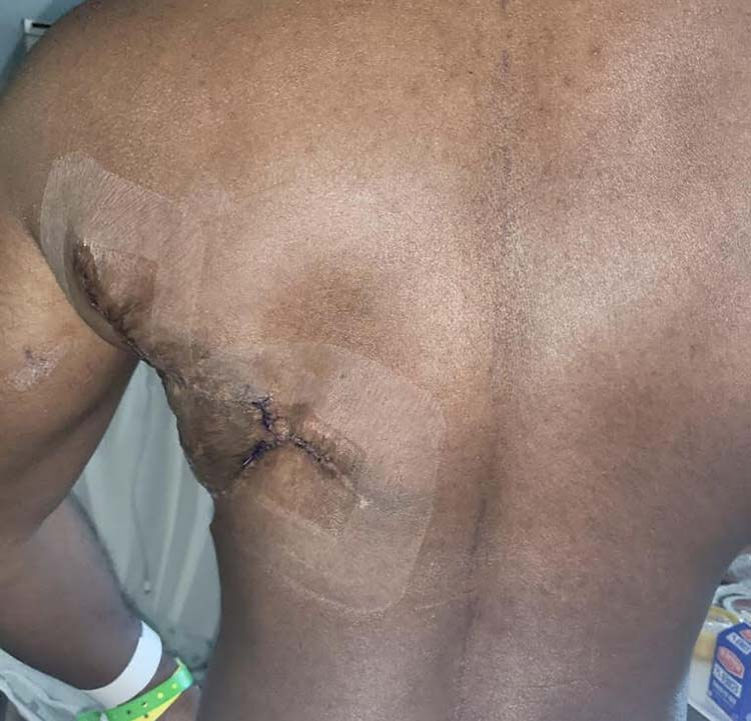
The patient’s donor site is shown 10 months postoperative following a left latissimus dorsi myocutaneous flap following one serial excision of the skin grafted donor site.

**Figure 6:**
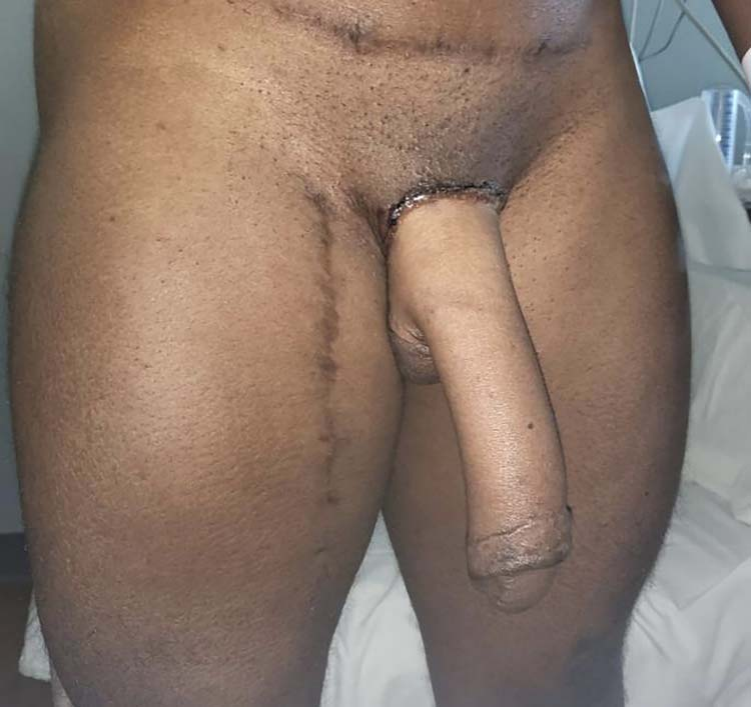
Patient is shown several weeks following placement of a two-rod malleable penile prosthesis.

Even though this procedure would lead to decreased urinary complications, it is more challenging to perform since the patient desired a phalloplasty with scrotoplasty and future testicular implant placement in an anatomic region occupied by his native introitus and vaginal canal (Fig. 1A and B). After careful consideration of the advantages and disadvantages of different flaps, the patient chose a musculocutaneous latissimus dorsi flap for his phalloplasty [10]. A disadvantage of this flap versus others is a lower sensory recovery because only one nerve, the lateral branch of the thoracodorsal nerve, is supplying it. The recipient artery chosen was the descending branch of the lateral femoral circumflex, the recipient vein was the greater saphenous vein and nerve anastomosis was a split lateral thoracodorsal nerve with one end anastomosed to the ilioinguinal nerve on the left (end to end with 9-0 nylon suture epineural sutures) and the clitoral nerve on the right (end to side) with epineural sutures using 9-0 nylon suture and an operative microscope. Cadaver nerve grafts from Axogen Inc., Alachua, FL, were used as interposition nerve grafts in both nerve anastomoses. Advantages include a more concealed donor site and a very sizeable tissue construction, although this means a skin graft on the donor site might be necessary, readily removed with serial excisions or tissue expansion if desired. A scrotoplasty was performed using the labia majora soft tissues, later used for testicular implants. This was created with bilateral V-Y random pattern labia majora flaps advanced inferiorly to just superior to the donor defect within the inferior labia majora. These are sutured in the midline and cephalad to the clitoral hood region. Careful attention was paid to maintain the vaginal canal appearance and capacity while constructing the phallus, especially during the microvascular portion of the procedure. The phallus was constructed, and the clitoral hood, clitoris and vaginal canal were not injured or modified (Fig. 2). Hegar dilators were useful for identification of the vaginal canal during the dissection to avoid injury to the vaginal canal and maintain adequate aperture (Fig. 3). Six months later after flap transfer, the patient had an insertion of bilateral testicular prosthesis above clitoral hood prior to penile implant placement (Fig. 4A and B). Serial excision of skin grafted donor site was followed by a left latissimus dorsi myocutaneous flap (Fig. 5). Successful insertion of penile prosthesis was performed at 8 months when the patient exhibited tactile sensation to two-thirds down his penile shaft from the base of his phallus. (Fig. 6).

### Case 2

**Figure 7:**
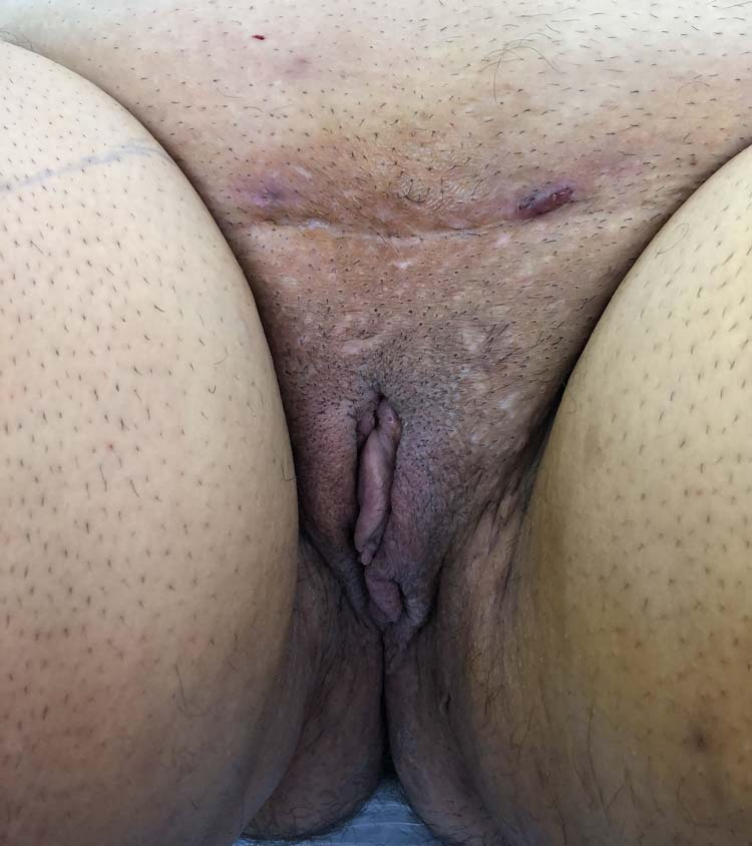
A 34-year-old trans man with history of hidradenitis suppurativa excisions prior to a tubed radial forearm flap phalloplasty with urethral lengthening and preservation of his reproductive organs and vaginal canal. Success full vaginal penetration, orgasm and recipient of vaginal penetration.

**Figure 8:**
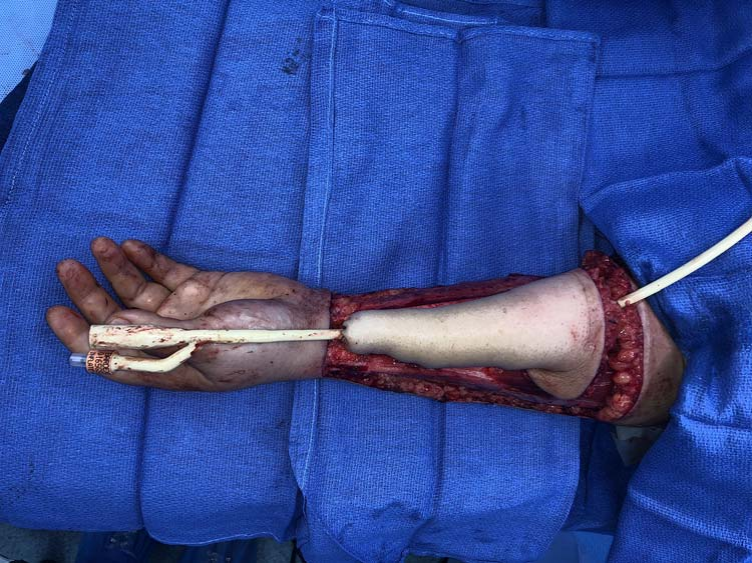
Tubed radial forearm flap for phalloplasty construction prior to flap transfer.

**Figure 9:**
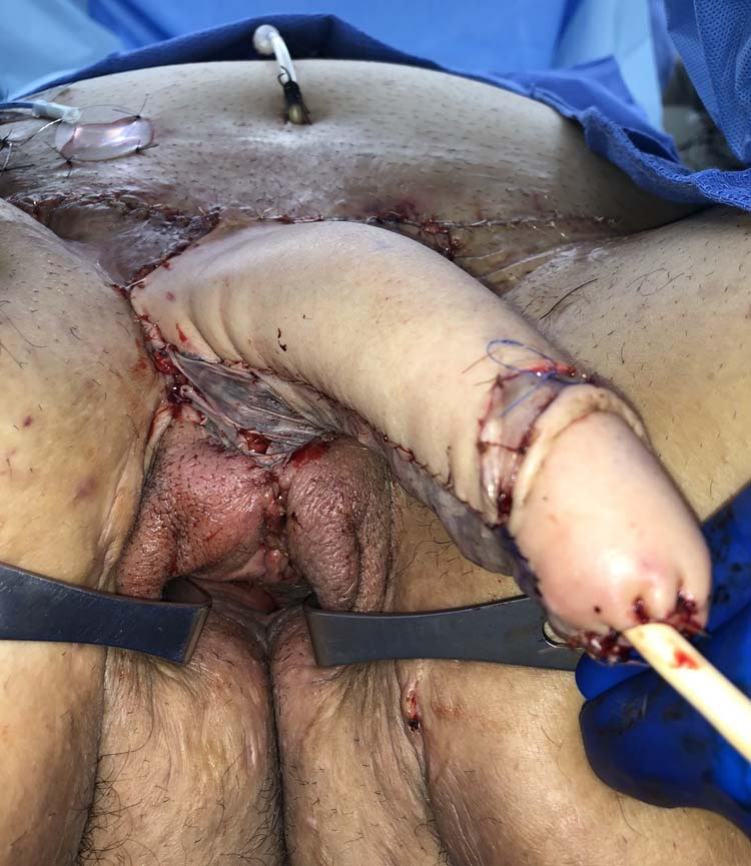
Patient shown immediately after radial forearm flap phalloplasty and urethroplasty with coronoplasty and suprapubic tube placement. A scrotoplasty was not desired and the vaginal canal with reproductive organs has been preserved.

**Figure 10:**
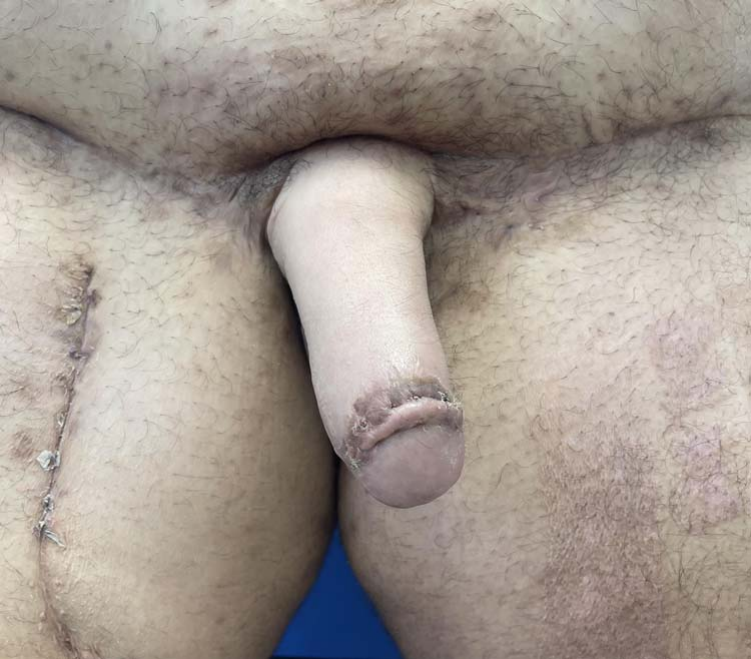
Patient is shown following placement of a two-rod silicone penile prosthesis and resection of his reproductive organs and vaginal canal. Successful vaginal penetration with orgasm.

Patient is a 34-year-old trans man who presented with a history of hidradenitis suppurativa and previous excisions in the abdominal wall and groin (Fig. 7). A tubed radial forearm flap phalloplasty with urethral lengthening and preservation of his reproductive organs, including his vaginal canal, due to desire for conception later was planned (Fig. 8). Urethral lengthening was performed with labia minora flaps and an anterior vaginal wall flap anteriorly based with urethral anastomosis to the tubed radial forearm flap. The anterior vaginal wall mucosal defect was left to heal so the canal would not be significantly narrowed. Recipient vessels for vasculature were to the descending branch of the lateral femoral circumflex artery and the saphenous vein for outflow. Nerve anastomosis was performed with the medial and lateral antebrachial nerves end to side to the clitoral nerve on the right and to both ilioinguinal nerves using an allograft nerve interposition for the clitoral nerve anastomosis. The natal clitoris was buried at the base of the phalloplasty construction (Fig. 9). Insertion of a penile prosthesis was later performed successfully at 3 years (Fig. 10). It is of note that the patient recognized he would have to discontinue testosterone therapy for 6 months prior to conception and later decided not to conceive, undergoing resection of his reproductive organs and vaginal canal 3 years after.

## DISCUSSION

The phallus flaps of both patients survived with no vascular compromise or partial flap loss. Skin grafts to flap donor site were required in both cases. Both patients described a significant reduction in their gender incongruence. Sensation was reported in their phallus construction two-thirds distally down their shaft on their last follow-up greater than 8 months and average of 22 months after surgical intervention. Both patients were able to maintain erogenous sensation from stimulation of their natal clitoris, exposed in one case and buried under flap construction in the other. Having penetrative vaginal intercourse with their partners was successful for both patients, before and after undergoing penile implant. The patient who underwent a radial forearm flap did have a urethral fistula, requiring urethral reconstruction with a buccal mucosa graft. He also decided to remove his reproductive organs and vaginal canal with scrotoplasty several years later.

The level of one’s gender incongruence solely depends on the individual suffering from it. For providers caring for this patient population, discussing the leading cause of the patient’s feelings of dysphoria to appropriately address it is extremely important. If the cause is the desire for chest masculinization or a phalloplasty, then surgical modification should ensue. The cause could be menses or simply the appearance of a vagina, though regardless of what the reason is, surgeons can assist the patient in this transition. The primary goal should always be to decrease the patient’s dysphoria so they feel more comfortable in the gender they identify with. Therefore, modifications to our proposed procedures should always be considered if within the capabilities of the operating surgeon.

A thorough conversation regarding realistic expectations with the patient regarding their desires as they transition needs to occur. Does the patient want to remove their reproductive organs if undergoing a phalloplasty? Does he desire a vaginectomy? Would he like to harvest his eggs, and is he aware of the costs associated? Would he like to undergo an oophorectomy at that time or preserve his ovaries until a later date? How important is urethral lengthening to them given the urinary complications that may result? If the vagina is to be removed, does the patient prefer a lengthened urethra or a perineal urethra? Once these questions are answered, the surgical plan is designed with the treating surgeon.

In cases where the vagina is preserved, the method with least complications is to maintain the native urethral orifice. Urethral lengthening requires modification of the anterior wall of the vagina, which may narrow the vaginal canal and risk urethral sequelae. The risks surrounding the preservation of reproductive structures and the need for their screening must be emphasized as well. In cases where the reproductive organs or vaginal canal with the cervix have been preserved, screening for cancer is best followed by both a urologist and gynecologist so that surveillance is adequately maintained by physicians knowledgeable of this particular anatomy. In this series, we have described surgical options during traditional phalloplasty that, given proper patient counseling, may be conducted so patient satisfaction may be achieved.

## CONFLICT OF INTEREST STATEMENT

None declared.

## CONSENT FOR PUBLICATION

Written informed consent was obtained from the patients for publication of this report and accompanying images.
